# The Exacerbation of Aging and Oxidative Stress in the Epididymis of *Sod1* Null Mice

**DOI:** 10.3390/antiox9020151

**Published:** 2020-02-11

**Authors:** Anaīs Noblanc, Alicia Klaassen, Bernard Robaire

**Affiliations:** 1 Department of Pharmacology and Therapeutics, McGill University, Montreal, QC H3G 1Y6, Canada; anaisnoblanc@hotmail.fr (A.N.); alicia.klaassen@mail.mcgill.ca (A.K.); 2 Department of Obstetrics and Gynecology, McGill University, Montreal, QC H4A 3J1, Canada

**Keywords:** aging, epididymis, spermatozoa, oxidative stress, reactive oxygen species, superoxide dismutase, 4-hydroxynonenal, 8-hydroxyguanosine

## Abstract

There is growing evidence that the quality of spermatozoa decreases with age and that children of older fathers have a higher incidence of birth defects and genetic mutations. The free radical theory of aging proposes that changes with aging are due to the accumulation of damage induced by exposure to excess reactive oxygen species. We showed previously that absence of the superoxide dismutase 1 (*Sod1*) antioxidant gene results in impaired mechanisms of repairing DNA damage in the testis in young *Sod1^−/−^* mice. In this study, we examined the effects of aging and the *Sod^−/−^* mutation on mice epididymal histology and the expression of markers of oxidative damage. We found that both oxidative nucleic acid damage (via 8-hydroxyguanosine) and lipid peroxidation (via 4-hydroxynonenal) increased with age and in *Sod1^−/−^* mice. These findings indicate that lack of SOD1 results in an exacerbation of the oxidative damage accumulation-related aging phenotype.

## 1. Introduction

Reactive oxygen species (ROS) generate chain reactions that can affect numerous biomolecules, including lipids, RNA, DNA, proteins [1,2,3,4]. The consequences of these biochemical reactions are part of the normal cellular function but, in excess, they can be deleterious and severely damage major cellular processes. We will focus on the impact of oxidative stress during aging in the male reproductive system and, specifically, in the epididymis. Most studies on the effects of aging on the epididymis were done using the Brown Norway rat model, a reliable model of aging [5,6,7,8]. The major characteristics of aging in the epididymis are a thickening of the basement membrane along the epididymis, a decreased integrity and functionality of the blood-epididymis barrier [9], a segment-dependent variation of each cell type distribution; in particular a decrease in the proportion of principal, clear, and basal cells and an increase in the number of halo cells in the entire epididymis was found [10]. Halo cells are immune cells and their increased number in the epididymal epithelium may reflect a lack of balance of the immune steady-state of the epididymis, in accordance with the proposed general hallmarks of aging [11].

Aging is a dynamic and continuous process with time-dependent physiological and molecular changes. Some of the major hallmarks of aging include changes in telomere length, genetic instability and an accumulation of epigenetic modifications, mitochondrial dysfunction, a decrease in the stem cell reserve, and chronic inflammation [11,12,13]. In 1956, a “Free Radical Theory of Aging” was proposed that states that aging is the consequence of an accumulation of oxidative damage on biomolecules [14,15].

In most species, aging is associated with an accumulation of oxidized biomolecules (proteins, nucleic acids, and lipids), resulting in the dysregulation of metabolism, with an increase in the leak of superoxide anions by the mitochondria and a decrease in the ability of cells to scavenge ROS and to repair oxidative damage. ROS play a major role in the aging process via their action on various signaling pathways, including insulin/insulin like growth factor 1 (IGF1), mammalian target of rapamycin kinase (mTOR) and AMP kinase (AMPK) [16,17,18,19,20,21]. The null mutation of *Igf1* in mice induces a shrinking of the epididymis from the corpus epididymidis to the vas deferens due to a major decrease in the sperm cell content of the tubule [22]. *Igf1* gene expression is induced in the epididymis of orchidectomized adult rats and is under an androgenic control [23].

ROS play essential roles in male reproduction. One of these is the production of disulfide bridges between cysteine thiol groups; this biochemical reaction is mandatory for the post-translational maturation of numerous proteins and various enzymatic reactions. During spermiogenesis, sperm DNA is compacted first through replacement of histones by transition proteins and then by cysteine-rich protamines [24,25]. Therefore, numerous disulfide bonds are generated when condensing sperm nucleus undergo 3D re-organization during spermatid elongation and spermatozoal maturation in the epididymis. Mice knocked out for the mitochondrial isoform of *Gpv4* (mGPX4) are infertile, due to non-motile spermatozoa with abnormal mid-piece and broken flagella [26,27]. Another form of this enzyme that is considered to play a major role in this process is the sperm nuclear isoform of GPX4 (snGPX4) [28,29,30,31]. The *snGpx4^−/−^* mice are viable and fertile, but their sperm chromatin shows a delay in chromatin condensation during maturation in the epididymis that is linked to a decrease in disulfide bridging in the caput epididymal sperm. Finally, the acrosome matrix is structured and held by numerous disulfide bridges and the inhibition of the disulfide isomerase enzymatic activity by sperm pre-treatment decreases the fertilization level during sperm-egg fusion in vitro [32].

ROS are also an essential component of the redox signaling pathways during sperm capacitation in the female tract. This process involves a complex group of sperm modifications to prepare the spermatozoa to undergo flagellar hyperactivity and the acrosome reaction, both necessary to the journey through the female tract and for sperm-egg recognition and fusion. Numerous reviews have provided excellent description of this process [33,34] and the role of ROS in this phenomenon [35,36,37].

However, despite the obvious need of ROS for sperm production, maturation, and function, spermatozoa are highly sensitive to oxidative damage. ROS can modify purine and pyrimidine bases, break nucleic acid backbone, and create covalent bonds intra-/inter-strands, and between nucleic acids and proteins [38]. The main target of ROS is guanine due to its lower redox potential, with the major by-product of the DNA oxidation being 7,8-dihydro-8-oxo-2’-deoxyguanosine (8-OHdG); this oxidative DNA damage can be lethal for cells. While spermatogonia and spermatocytes have extensive DNA repair machinery, condensing spermatids and spermatozoa lose their DNA repair capacity [39,40,41]. 

Lipids are also targets of ROS, especially sterols and polyunsaturated fatty acids; these lipids affect the fluidity of the sperm cytoplasmic membrane. Lipid peroxidation can result in the generation of numerous toxic side products (alkanes, aldehydes, and acids), including 4-hydroxy-2-nonenal (4-HNE) which is highly cytotoxic, a molecule that is commonly used as a marker of lipid peroxidation. Lipid peroxidation decreases the fluidity of cell membranes, a major concern for sperm motility and ability to undergo capacitation and sperm-egg fusion [42,43].

Redox balance must be tightly maintained to limit the negative effects of ROS on reproductive tissues and spermatozoa, particularly during epididymal maturation when sperm are devoid of the ability to react to their environment. Consequently, the epididymal epithelium must play a protective role. A wide array of antioxidant enzymes is present in the epididymis, both in the epithelium and the luminal fluid; these include superoxide dismutases (SODs), catalase, glutathione transferases, glutathione peroxidases, and peroxiredoxins [44].

SODs are a family of metalloproteins composed of three different members, located in different cellular compartments and associated with different metal ions. SOD1, or Cu/ZnSOD is located in the intermembrane space of mitochondria, in the nucleus and in the cytoplasm [45]. Mutations of the *Sod1* gene have been associated with human pathologies such as amyotrophic lateral sclerosis [46]. Male mice lacking the ability to synthesize SOD1 are fertile but in vitro studies demonstrate that several characteristics of spermatozoa from *Sod1*^−/−^ mice are affected, including higher lipid peroxidation, decreased motility, impaired capacitation ability, and a low fertilizing ability in in vitro fertilization [47]. 

*Sod1*^−/−^ mice with a C57Bl/6 genetic background provide an interesting rodent model to test the free radical theory of aging and the mechanisms involved in the aging process. The phenotype of C57Bl/6 mice during aging is well characterized, as this animal model is often used for aging studies [48,49]. Despite a relatively normal phenotype and health during development and young adulthood, *Sod1*^−/−^ mice have a reduced lifespan of 20.8 ± 0.7 (mean ± S.D.) months compared to the lifespan of wild-type mice of 29.8 ± 2.1 months [50]. The absence of SOD1 leads to extensive oxidative damage in various tissues including the intestine, liver, ovary, and testis [46,47,48,49,50,51,52,53] as early as 3 months of age and even in fetal fibroblasts [54]. In previous studies of the effects of the lack of SOD1 on the male reproductive system, we reported an impact on the male germline with a decreased ability to scavenge ROS, an increased number of oxidized spermatozoa, and a reduced DNA repair machinery [53]. In the present study, we examine the effects of aging on the epididymis of wild-type and *Sod1*^−/−^ mice.

## 2. Materials and Methods

### 2.1. Animal Model

B6.129S7-Sod1^tm1Leb^/DnJ mice were purchased from the Jackson Laboratory (#003881, Bar Harbor, ME, USA). They were backcrossed with C57Bl/6NJ mice (#005304, Bar Harbor, ME, USA) and bred in-house at McGill University in the McIntyre Comparative Medicine and Animal Resources Centre. Mice were kept on a 12L:12D cycle; food and water were provided *ad libitum*, and the temperature was maintained at 22 °C. Young (3 to 4-month-old) and aged (18-month-old) wild-type (WT) and *Sod1* null (*Sod1*^−/−^) mutants mice were selected for this study. The number of mice in each group was from 3 to 6. Mice were euthanized by CO_2_ asphyxiation followed by cervical dislocation. Both epididymides were harvested. All animal studies were conducted in accordance with the principles and procedures outlined in the Guide to the Care and Use of Experimental Animals prepared by the Canadian Council on Animal Care (McGill Animal Care Committee protocol #4687).

### 2.2. Tissue Fixation

Epididymides were fixed using Modified Davidson’s fluid (12% formaldehyde, 15% ethanol, 5% glacial acetic acid) for 8 h at 4 °C [55]. Tissues were then washed in 70% ethanol, dehydrated, embedded in paraffin and sectioned at 4 µm at the Goodman Cancer Research Center Histology Facility (McGill University, Montreal, QC, Canada).

### 2.3. Histology

Sections were stained with toluidine blue. Tissues were deparaffinized (Histoclear; Diamed Inc., Mississauga, ON Canada), rehydrated and stained with 0.1% (w/v) Toluidine Blue O (#198161, Sigma) in 0.25% acid alcohol (HCl) solution. The slides were mounted with Permount™ mounting medium (Fisher Scientific, Montreal, QC, Canada). Sections were observed under bright field microscopy using a Leica LB2 microscope at 400× magnification.

### 2.4. Immunofluorescence

Tissue sections were deparaffinized and rehydrated. Heat-induced antigen retrieval in 10mM sodium citrate, pH 6.0, 0.05% Tween 20 was done prior to immunostaining for 4-hydroxynonenal (4-HNE) protein adducts and non-receptor tyrosine kinase SRC (SRC), but not for 8-hydroxyguanosine (8-OHG). The slides were washed in Tris Buffered Saline 1×, 0.02% Tween 20 (TBS-T) and were incubated in TBS-T, 5% normal goat serum (NGS) at room temperature for 30 min. Tissue sections were incubated overnight at 4 °C with the primary antibodies diluted in TBS-T, 1% NGS (Table 1). They were washed twice in TBS-T and incubated at room temperature for 1 hour with the secondary antibodies diluted in TBS-T, 1% NGS, 1 µg/mL4’,6-diamidine-2’-phenylindole dihydrochloride (DAPI). After one wash in TBS-T and two washes in TBS 1X, slides were mounted with PermaFluor™ mounting medium (ThermoFisher Scientific, Montreal, QC, Canada). For the negative controls, the primary antibodies were omitted.

### 2.5. Immunofluorescence Imaging and Quantification 

Imaging of each full section was done with the Opera Phenix™ high-content screening system and Harmony software (Perkin Elmer, Montreal, QC, Canada) with a field overlap of 5%, at a 40× magnification. The images were further analyzed using Columbus™ system (Perkin Elmer, Montreal, QC, Canada) to quantify the signal of the immunofluorescent staining. Analyses were applied which recognized DAPI as a nucleus marker and SRC as a cytoplasmic marker to segment the cells for quantification of fluorescence intensity in each cellular compartment. The 4-HNE and 8-OHG intensities were used to determine oxidative damage in membranes and DNA, respectively. Analyses were done separately in the caput, corpus and cauda epididymides, as well as in the epithelium and in the interstitial cells (endothelial, smooth muscle, and conjunctive cells).

### 2.6. Statistical Analyses

All graphical data are represented as the means ± SEM. Prior to analysis, data were determined to be normally distributed. All data were then analyzed using a 3-way analysis of variance ANOVA with genotype, epididymal segment and age as variables, followed by Tukey’s multiple comparisons test. All statistical analyses were done with the software Prism8 v8.0.1 (GraphPad Software, La Jolla, CA, USA). Differences among samples were considered to be significant when the *p* < 0.05.

## 3. Results

### 3.1. Histology

**Figure 1:**
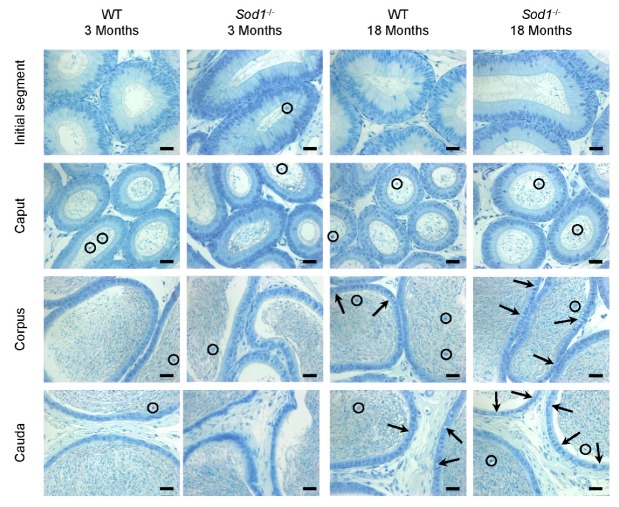
The histology of the epididymis is affected by aging in wild-type mice and even more so in *Sod1^−/−^* mice. Representative pictures of epididymal sections (initial segment, caput, corpus, and cauda epididymides) stained with toluidine blue from 3-month-old and 18-month-old wild-type and *Sod1^−/−^* mice. An increase in luminal round cells (circles) and in the number and size of clear vesicles (arrows) were observed in the epididymal epithelium of the corpus and cauda epididymides (n = 3). Scale bar: 40 µm.

Representative sections of the initial segment, caput, corpus and cauda regions of the epididymis of young and old wild-type and *Sod^−/−^* mice are shown in Figure 1. No major differences were observed between young *Sod1^−/−^* and WT mice (3 months) with respect to either the epididymal tubule histology (Figure 1) or the interstitial tissue histology (smooth muscle cells, conjunctive tissue, vascular system) (data not shown). In old (18 months) *Sod1^−/−^* and WT mice distinctive features included an increase in the tubule diameter all along the epididymis, a decrease in the height of epididymal cells, as well as an accumulation of spermatozoa in the initial segments, where they are usually absent. There was also an increase in luminal round cells (circles). Further, we observed the presence of large vacuoles in some epithelial cells in the corpus and cauda epididymides (arrows). The thickness of the myoid layer surrounding the epididymal tubule was increased only in the cauda epididymidis. Among these features, only two were enhanced by the absence of the SOD1 protein in this tissue. The accumulation of cellular vacuoles in the cauda epididymidis was increased in old *Sod1^−/−^* mice compared to old WT mice. Moreover, the thickening of the myoid layer was greater in old *Sod1^−/−^* mice than in old WT mice, but this is also visible in cauda epididymidis of young *Sod1^−/−^* mice. In one old *Sod1^−/−^* mouse, the accumulation of an amorphous matter in the epididymal lumen of the caput, corpus, and cauda epididymides was associated with round cells only in the cauda epididymides [53]. Thus, overall our observations indicate that aging affects the histology of the epididymis and this phenotype is enhanced by the absence of the SOD1 protein.

### 3.2. DNA Oxidation

**Figure 2:**
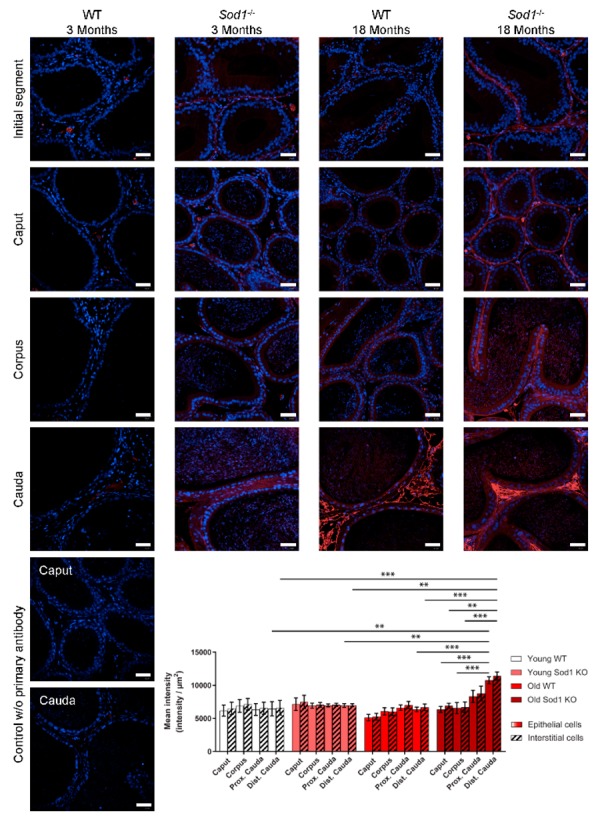
Aging induces an accumulation the nucleic acid damage in the epididymis (initial segment, caput, corpus, and cauda epididymides) which is enhanced in *Sod1^−/−^* mice. Representative pictures of sections of the epididymis from 3-month-old and 18-month-old wild-type and *Sod1^−/−^* mice after immunostaining of oxidized nucleic acids (8-OHG, red); the nuclei are counterstained with DAPI (blue). Immunostaining negative controls (no primary antibody) are displayed for the caput and cauda epididymides. Scale bar: 40 µm. 8-OHG staining was quantified separately in the epididymal epithelium (clear histograms) and in the interstitial tissue (dashed histograms). ** *p* < 0.01, *** *p* < 0.001 (3-way ANOVA, n = 4–5).

DNA oxidation was assessed by quantitative immunostaining of the oxidized guanine in the nucleus (8-OHdG). Immunofluorescent staining was detected in both epithelial and interstitial cells of the different regions of the epididymis (initial segment, caput, corpus, and cauda) (Figure 2). In 18-month-old mice, staining was more intense in the cauda epididymidis than in the rest of the tissue; further, this increase was more pronounced in the distal cauda epididymidis than in the proximal cauda epididymidis. Quantification of nuclear 8-OHdG immunofluorescent staining (Figure 2 bar graph) indicated that the major increase in DNA oxidation was observed in the cauda epididymidis of 18-month-old *Sod1^−/−^* mice. Statistical analysis by 3-way ANOVA (age, genotype, region, Table 2) revealed that the main source of variation in the DNA oxidation levels was the *Sod1^−/−^* genotype; in addition, there were interactions between epididymal regions and age and between age and genotype.

### 3.3. Lipid Peroxidation

**Figure 3:**
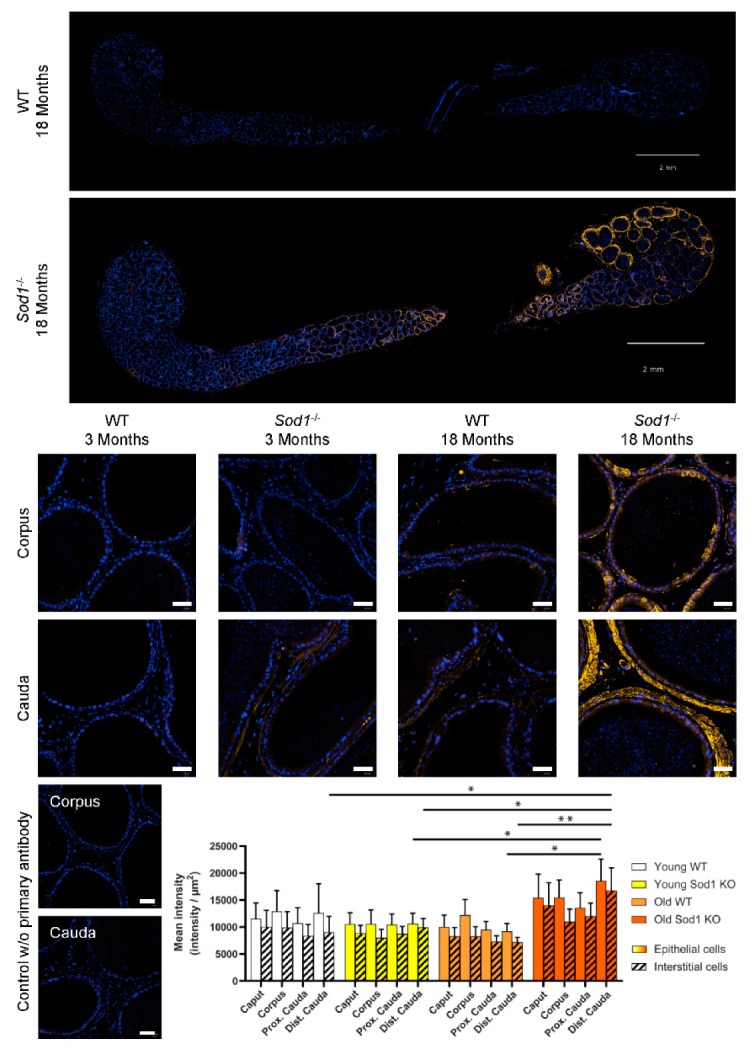
Lipid peroxidation as assessed by 4-HNE immunostaining is increased in the corpus and cauda epididymides with aging and this phenotype is enhanced in the absence of SOD1 expression. Representative pictures of whole epididymis sections of 18-month-old wild-type and *Sod1^−/−^* mice after the immunostaining of the 4-HNE (yellow) and the counterstaining of the nucleus (DAPI, blue). Scale bar: 2 mm. Observations have been carried out in epididymis sections of 3-month-old and 18-month-old wildtype and *Sod1^−/−^* mice. Staining was strongest in the corpus and cauda epididymides on the apical membrane of epithelial cells (arrow) and on intracellular membranes (arrow head). The negative controls (no primary antibody) for immunostaining are displayed for the corpus and cauda epididymides. Scale bar: 40 µm. The 4-HNE staining has been quantified in the epididymal epithelium (clear histograms) and in the interstitial tissue (dashed histograms) separately. * *p* < 0.05; ** *p* < 0.01 (3-way ANOVA, n = 4–5).

Lipid peroxidation was evaluated by immunostaining of 4-hydroxynonenal (4-HNE) protein adducts, a by-product of lipid peroxidation. Longitudinal sections of the epididymides of 18-month-old WT and *Sod1^−/−^* mice revealed major differences in 4-HNE staining intensity and its regional distribution (Figure 3). Staining was absent in the initial segment and the caput epididymidis and was found almost exclusively in the corpus and the cauda epididymides. Further, in 18-month-old *Sod1^−/−^* mice, it appeared to be higher in the distal than in the proximal cauda epididymidis. The 4-HNE staining was detected mainly on cytoplasmic membranes, particularly on the apical membranes; in highly stained cells, it was also visible on intracellular and nuclear membranes. Quantitative analyses were done for staining on all cell surfaces. In the caput epididymidis, staining was indistinguishable to that seen in the control sections without primary antibody. The 3-way ANOVA analysis on quantitative data clearly indicated that there was a significant difference between the WT and *Sod1^−/−^* genotypes and that this effect was amplified by the age of the mice (Table 2).

## 4. Discussion

Various theories have been proposed to explain the complex physiological and molecular modifications that occur during aging [13]. Among the potential causes and/or consequences of aging are an increase in oxidative stress and the accumulation of oxidative damage on many cellular components. Thus, to study the effects and mechanisms of aging in the male reproductive system, we have investigated various animal models, particularly rats and mice, with or without a genetically induced oxidative stress [53,56]. In this study, we analyzed the consequences of aging and oxidative stress in the epididymis, the site where spermatozoa become mature, using *Sod^−/−^* mice. *Sod1^−/−^* mice have a decreased ability to protect against ROS attack, leading to an accumulation of oxidative damage even in young animals, and have a reduced lifespan [46,47,48,49]. 

We analyzed the histology of the epididymis of 3- and 18-month-old wild-type and *Sod1^−/−^* mice. We identified some features that are indicative of a decline in epididymis structure and function with aging. The most obvious phenotypic change observed in old WT and *Sod1^−/−^* mice is the appearance of vacuoles in the epididymal epithelial cells, selectively in the corpus and the cauda epididymides. This feature was described previously in the corpus and proximal cauda epididymides of old Brown Norway rats [10]. The vacuoles found in principal cells were identified as giant lysosomes and lipid droplets, suggesting that the digestion/recycling system and/or the intracellular trafficking are dysregulated during aging. Further analyses in these old Brown Norway rats demonstrated a variation of the expression of glutathione S-transferases, enzymes that play an important role in detoxification of electrophiles. Thus, it would be interesting to study the effects of aging on the expression of genes/proteins in the epididymis of the aging *Sod1^−/−^* mice to determine if the same process occurs in mice. 

Interestingly, we observed an increase in the number of round cells in the epididymal lumen of old mice compared to young mice; the nature of these cells is not clear. These cells were identified as round spermatids in some animal models of spermatogenic arrest but previous analyses of the testis in *Sod1^−/−^* mice did not reveal this phenotype [49]. Chronic and systemic inflammation is a hallmark of aging [11,12]. The increase in the number of halo cells (resident immune cells of the epididymis) along the epithelium during aging [57], in conjunction with the demonstration of dendritic cells [58], indicates a close interaction between the immune system and the epididymis. Therefore, it is interesting to speculate that these intraluminal round cells could be infiltrating immune cells, passed through the less impermeable blood-epididymis barrier of old animals [9]. An analysis of the blood-epididymis barrier and of the potential immune markers on the intraluminal round cells would be necessary to test this hypothesis,

The increase in the tubule diameter along the epididymis in old (18 month) *Sod1^−/−^*, accompanied by a decrease in epithelial cell height, is consistent with previous observations in C57Bl/6NJ mice [53]. Surprisingly, opposite histological features of aging have been observed in old Brown Norway rats [6]. In the hamster, the diameter of the lumen also decreases whereas epithelial height does not change between young and old animals [59]. 

Spermatozoa were present consistently in the lumen of the initial segment of the epididymis of young and old *Sod1^−/−^* mice compared to wild-type animals. This may be due to a decrease in the ability of the epididymis to transport spermatozoa. This transport is dependent on peristaltic contractions of the smooth muscle layer surrounding the tubule, pressure from the testicular fluid and new spermatozoa, and the movement of stereocilia at the apical pole of epithelial cells [60]. Another indication that the ability of the epididymis to transport spermatozoa is affected by aging is the increase in the smooth muscle layer in the distal cauda epididymidis where spermatozoa are stored between two ejaculations.

A progressive increase in the nucleic acid oxidation in the lumen of the tubule, from the caput epididymidis to the distal cauda epididymidis, was observed in 18-month-old mice, but not in 3-month-old wild-type mice. This phenomenon could be due to the increased quantity of spermatozoa contained in the lumen as long as they travel down the epididymis and to the increased generation of ROS by spermatozoa during aging [61].

Finally, we assessed 4-HNE immunofluorescent staining as a marker of lipid peroxidation. Lipid peroxidation appeared to be absent in the initial segment and the caput epididymidis and was only visible in the corpus and the cauda epididymidis. The staining was low in 3-month-old mice and increased with aging. As for the DNA oxidation, it increased along the tubule. The apical membranes of epithelial cells appeared to be more sensitive to lipid peroxidation. The reasons for the apical susceptibility to oxidative damage is unknown. It could be due to the direct contact with the luminal fluid and spermatozoa, a potential source of ROS, or to the high activity of this membrane (exocytosis, endocytosis, sensing of the luminal contents) which generates numerous reactions, another possible source of ROS. 

The absence of SOD1 in the mouse epididymis did not affect overall oxidation as assessed by markers of DNA and lipid oxidation in 3-month-old mice. Other members of the SOD family may compensate for this loss and the repair machinery of the various cell components seems able to deal with the low level of oxidized biomolecules. However, oxidized nucleic acids and peroxidized lipids increased strikingly in the epididymal tissue of 18-month-old *Sod1^−/−^* mice, even compared to the old wild-type mice. Histological analyses revealed an increase in the quantity and size of vacuoles in the epididymal epithelial cells of the corpus and the cauda epididymides and an increase in the thickening of the myoid cell layer in the distal cauda epididymidis in old *Sod1^−/−^* mice compared to old wild-type mice. Thus, there is clearly a worsening of the aging phenotype in the mouse epididymis in the absence of SOD1. These findings suggest that *Sod1*^−/−^ mice constitute a valuable model for better understanding aging in the epididymis.

## 5. Conclusions

Aging is a progressive biological process that is characterized by an accumulation of various physiological and molecular changes, including an increase in oxidative damage to different biomolecules, leading to cell and tissue dysfunctions. In this study, we evaluated the impact on the epididymis of removing SOD1 using *Sod1*^−/−^ mice. We demonstrated that, using this rodent model, the aging phenotype is exacerbated by increased oxidative damage.

## Figures and Tables

**Table 1:** List of antibodies used for immunofluorescence experiments.

Target	Conjugation	Clonality	Dilution	Company	Catalog
Primary Antibodies					
8-OHG (DNA/RNA)	None	Mouse Monoclonal (15A3)	1/1000	Novus Biologicals	NB110-96878
4-HNE	None	Rabbit polyclonal	1/500	Abcam	ab46545
SRC, C-term	None	Mouse monoclonal	1/250	Santa Cruz	sc8056
Secondary Antibodies					
Mouse whole IgG	Alexa Fluor 488	Goat polyclonal	1/500	Invitrogen	a11029
Rabbit whole IgG	Alexa Fluor 546	Goat polyclonal	1/1000	Invitrogen	a11010
Mouse whole IgG	Alexa Fluor 633	Goat polyclonal	1/2000	Invitrogen	a21046

8-hydroxyguanosine (8-OHG), 4-hydroxynonenal (4-HNE), proto-oncogene tyrosine-protein kinase (SRC).

**Table 2:** Statistical analysis of the staining quantification. The 3-way ANOVA was followed by Tukey’s multiple comparisons test performed using GraphPad Prism version 8.0.1 for Windows, GraphPad Software, San Diego, CA, USA.

Staining	8-Hydroxyguanine			4-Hydroxynonenal		
Source of Variation	% of Total Variation	*p* Value	Significance	% of Total Variation	*p* Value	Significance
Region	10.23	0.0122	*	5.78	0.3546	ns
Age	1.07	0.1604	ns	2.18	0.0880	ns
Genotype	14.00	<0.0001	****	4.47	0.0153	*
Region × Age	12.50	0.0030	**	0.31	0.9997	ns
Region × Genotype	5.38	0.1988	ns	1.84	0.9251	ns
Age × Genotype	5.37	0.0020	**	8.69	0.0008	***
Region × Age × Genotype	4.63	0.2897	ns	1.06	0.9833	ns

* *p* < 0.05, ** *p* < 0.01, *** *p* < 0.001, **** *p* < 0.0001.

## References

[1] Collin F. (2019). Chemical Basis of Reactive Oxygen Species Reactivity and Involvement in Neurodegenerative Diseases. Int. J. Mol. Sci..

[2] Ylä-Herttuala S. (1999). Oxidized LDL and Atherogenesis. Ann. New York Acad. Sci..

[3] Stadtman E.R., Levine R.L. (2000). Protein Oxidation. Ann. New York Acad. Sci..

[4] Marnett L.J. (2000). Oxyradicals and DNA Damage. Carcinogenesis.

[5] Wright W.W., Fiore C., Zirkin B.R. (1993). The Effect of Aging on the Seminiferous Epithelium of the Brown Norway Rat. J. Androl..

[6] Wang C., Hikim A.S., Ferrini M., Bonavera J.J., Vernet L., Leung A., Lue Y.-H., Gonzalez-Cadavid N.F., Swerdloff R.S. (2002). Male Reproductive Ageing: Using the Brown Norway Rat as a Model for Man. Novartis Found. Symp..

[7] Robaire B., Robaire B., Hinton B.T. (2002). Aging of the Epididymis. The Epididymis: From Molecules to Clinical Practice.

[8] Beattie M., Adekola L., Papadopoulos V., Chen H., Zirkin B. (2015). Leydig Cell Aging and Hypogonadism. Exp. Gerontol..

[9] Levy S., Robaire B. (1999). Segment-Specific Changes with Age in the Expression of Junctional Proteins and the Permeability of the Blood-Epididymis Barrier in Rats. Boil. Reprod..

[10] Serre V. (1998). Segment-Specific Morphological Changes in Aging Brown Norway Rat Epididymis. Boil. Reprod..

[11] López-Otín C., Blasco M.A., Partridge L., Serrano M., Kroemer G. (2013). The Hallmarks of Aging. Cell.

[12] McHugh D., Gil J. (2018). Senescence and Aging: Causes, Consequences, and Therapeutic Avenues. J. Cell Biol..

[13] Lipsky M.S., King M. (2015). Biological Theories of Aging. Dis. Mon..

[14] Harman D. (1956). Aging: A Theory Based on Free Radical and Radiation Chemistry. J. Gerontol..

[15] Terman A., Brunk U.T. (2006). Oxidative Stress, Accumulation of Biological ’Garbage’, and Aging. Antioxid. Redox Signal..

[16] Sarbassov D.D., Sabatini D.M. (2005). Redox Regulation of the Nutrient-Sensitive Raptor-mTOR Pathway and Complex. J. Boil. Chem..

[17] Yoshida S., Hong S., Suzuki T., Nada S., Mannan A.M., Wang J., Okada M., Guan K.-L., Inoki K. (2011). Redox Regulates Mammalian Target of Rapamycin Complex 1 (mTORC1) Activity by Modulating the TSC1/TSC2-Rheb GTPase Pathway. J. Boil. Chem..

[18] Ramírez-Rangel I., Bracho-Valdés I., Vázquez-Macías A., Carretero-Ortega J., Reyes-Cruz G., Vazquez-Prado J. (2011). Regulation of mTORC1 Complex Assembly and Signaling by GRp58/ERp57. Mol. Cell. Boil..

[19] Wang Q., Liang B., Shirwany N.A., Zou M.-H. (2011). 2-Deoxy-D-Glucose Treatment of Endothelial Cells Induces Autophagy by Reactive Oxygen Species-Mediated Activation of the AMP-Activated Protein Kinase. PLOS ONE.

[20] Chandrasekaran A., Idelchik M.D.P.S., Melendez J.A. (2017). Redox Control of Senescence and Age-Related Disease. Redox Biol..

[21] Weichhart T. (2018). MTOR as Regulator of Lifespan, Aging, and Cellular Senescence: A Mini-Review. Gerontology.

[22] Baker J., Hardy M.P., Zhou J., Bondy C., Lupu F., Bellvé A.R., Efstratiadis A. (1996). Effects of an Igf1 Gene Null Mutation on Mouse Reproduction. Mol. Endocrinol..

[23] Hamzeh M., Robaire B. (2010). Identification of Early Response Genes and Pathway Activated by Androgens in the Initial Segment and Caput Regions of the Regressed Rat Epididymis. Endocrinology.

[24] Balhorn R. (2007). The Protamine Family of Sperm Nuclear Proteins. Genome Boil..

[25] Ward W.S. (2018). Organization of Sperm DNA by the Nuclear Matrix. Am. J. Clin. Exp. Urol..

[26] Maiorino M., Roveri A., Benazzi L., Bosello V., Mauri P., Toppo S., Tosatto S.C.E., Ursini F. (2005). Functional Interaction of Phospholipid Hydroperoxide Glutathione Peroxidase with Sperm Mitochondrion-associated Cysteine-rich Protein Discloses the Adjacent Cysteine Motif as a New Substrate of the Selenoperoxidase. J. Boil. Chem..

[27] Ursini F. (1999). Dual Function of the Selenoprotein PHGPx During Sperm Maturation. Science.

[28] Conrad M., Moreno S.G., Sinowatz F., Ursini F., Kölle S., Roveri A., Brielmeier M., Wurst W., Maiorino M., Bornkamm G.W. (2005). The Nuclear Form of Phospholipid Hydroperoxide Glutathione Peroxidase Is a Protein Thiol Peroxidase Contributing to Sperm Chromatin Stability. Mol. Cell. Boil..

[29] Noblanc A., Peltier M., Damon-Soubeyrand C., Kerchkove N., Chabory E., Vernet P., Saez F., Cadet R., Janny L. (2012). and Pons-Rejraji, H. et al. Epididymis Response Partly Compensates for Spermatozoa Oxidative Defects in snGPx4 and GPx5 Double Mutant Mice. PLoS ONE.

[30] Pfeifer H. (2001). Identification of a Specific Sperm Nuclei Selenoenzyme Necessary for Protamine Thiol Cross-Linking during Sperm Maturation. FASEB J..

[31] Puglisi R., Maccari I., Pipolo S., Conrad M., Mangia F., Boitani C. (2012). The Nuclear Form of Glutathione Peroxidase 4 Is Associated with Sperm Nuclear Matrix and Is Required for Proper Paternal Chromatin Decondensation at Fertilization. J. Cell. Physiol..

[32] Ellerman D.A., Myles D.G., Primàkoff P. (2006). A Role for Sperm Surface Protein Disulfide Isomerase Activity in Gamete Fusion: Evidence for the Participation of ERp57. Dev. Cell.

[33] Gangwar D., Atreja S. (2015). Signalling Events and Associated Pathways Related to the Mammalian Sperm Capacitation. Reprod. Domest. Anim..

[34] Molina L.C.P., Luque G.M., Balestrini P.A., Marín-Briggiler C.I., Romarowski A., Buffone M.G. (2018). Molecular Basis of Human Sperm Capacitation. Front. Cell Dev. Boil..

[35] De Lamirande E., O’Flaherty C. (2008). Sperm Activation: Role of Reactive Oxygen Species and Kinases. Biochim. Biophys. Acta (BBA) Proteins Proteom..

[36] De Lamirande E., Zini A., Gabriel M.C. (2012). Human Sperm Chromatin Undergoes Physiological Remodeling During in Vitro Capacitation and Acrosome Reaction. J. Androl..

[37] O’Flaherty C., De Lamirande E., Gagnon C. (2006). Positive Role of Reactive Oxygen Species in Mammalian Sperm Capacitation: Triggering and Modulation of Phosphorylation Events. Free. Radic. Boil. Med..

[38] Jena N.R. (2012). DNA Damage by Reactive Species: Mechanisms, Mutation and Repair. J. Biosci..

[39] Iguchi N., Tobias J.W., Hecht N.B. (2006). Expression Profiling Reveals Meiotic Male Germ Cell mRNAs that Are Translationally Up- and Down-Regulated. Proc. Natl. Acad. Sci..

[40] Hao S.-L., Ni F.-D., Yang W.-X. (2019). The Dynamics and Regulation of Chromatin Remodeling during Spermiogenesis. Gene.

[41] Cavé T., Desmarais R., Lacombe-Burgoyne C., Boissonneault G. (2019). Genetic Instability and Chromatin Remodeling in Spermatids. Genes.

[42] Aitken R.J., Clarkson J.S., Fishel S. (1989). Generation of Reactive Oxygen Species, Lipid Peroxidation, and Human Sperm Function. Boil. Reprod..

[43] Gomez E., Irvine D.S., Aitken R.J. (1998). Evaluation of a Spectrophotometric Assay for the Measurement of Malondialdehyde and 4-hydroxyalkenals in Human Spermatozoa: Relationships with Semen Quality and Sperm Function. Int. J. Androl..

[44] O’Flaherty C. (2019). Orchestrating the Antioxidant Defenses in the Epididymis. Andrology.

[45] Mccord J.M., Fridovich I. (1970). The Utility of Superoxide Dismutase in Studying Free Radical Reactions. II. The Mechanism of the Mediation of Cytochrome c Reduction by a Variety of Electron Carriers. J. Boil. Chem..

[46] Rosen D.R., Siddique T., Patterson D., Figlewicz D.A., Sapp P., Hentati A., Donaldson D., Goto J., O’Regan J.P., Deng H.X. (1993). Mutations in Cu/Zn Superoxide Dismutase Gene Are Associated with Familial Amyotrophic Lateral Sclerosis. Nature.

[47] Tsunoda S., Kawano N., Miyado K., Kimura N., Fujii J. (2012). Impaired Fertilizing Ability of Superoxide Dismutase 1-Deficient Mouse Sperm During in Vitro Fertilization1. Boil. Reprod..

[48] Brayton C.F., Treuting P.M., Ward J.M. (2012). Pathobiology of Aging Mice and GEM: Background Strains and Experimental Design. Vet. Pathol..

[49] Pettan-Brewer C., Treuting P.M. (2011). Practical Pathology of Aging Mice. Pathobiol. Aging Age Relat. Dis..

[50] Elchuri S., Oberley T.D., Qi W., Eisenstein R.S., Jackson Roberts L., Van Remmen H., Epstein C.J., Huang T.-T. (2005). CuZnSOD Deficiency Leads to Persistent and Widespread Oxidative Damage and Hepatocarcinogenesis Later in Life. Oncogene.

[51] Kruidenier L. (2003). Attenuated Mild Colonic Inflammation and Improved Survival from Severe DSS-Colitis of Transgenic Cu/Zn-SOD Mice. Free. Radic. Boil. Med..

[52] Matzuk M.M., Dionne L., Guo Q., Kumar T.R., Lebovitz R.M. (1998). Ovarian Function in Superoxide Dismutase 1 and 2 Knockout Mice. Endocrinology.

[53] Selvaratnam J.S., Robaire B. (2016). Effects of Aging and Oxidative Stress on Spermatozoa of Superoxide-Dismutase 1- and Catalase-Null Mice1. Boil. Reprod..

[54] Huang T.-T., Yasunami M., Carlson E.J., Gillespie A.M., Reaume A.G., Hoffman E.K., Chan P.H., Scott R.W., Epstein C.J. (1997). Superoxide-Mediated Cytotoxicity in Superoxide Dismutase-Deficient Fetal Fibroblasts. Arch. Biochem. Biophys..

[55] Latendresse J.R., Warbrittion A.R., Jonassen H., Creasy D.M. (2002). Fixation of Testes and Eyes Using a Modified Davidson’s fluid: Comparison with Bouin’s Fluid and Conventional Davidson’s Fluid. Toxicol. Pathol..

[56] Selvaratnam J., Paul C., Robaire B. (2015). Male Rat Germ Cells Display Age-Dependent and Cell-Specific Susceptibility in Response to Oxidative Stress Challenges1. Boil. Reprod..

[57] Serre V. (1999). Distribution of Immune Cells in the Epididymis of the Aging Brown Norway Rat Is Segment-Specific and Related to the Luminal Content. Boil. Reprod..

[58] Da Silva N., Barton C.R. (2016). Macrophages and Dendritic Cells in the Post-Testicular Environment. Cell Tissue Res..

[59] Calvo A., Pastor L.M., Roca J., Martínez E., Vázquez J.M. (1999). Age-Related Changes in the Hamster Epididymis. Anat. Rec..

[60] Robaire B., Hinton B., Plant T., Zeleznik A. (2014). The Epididymis. Knobil and Neill’s Physiology of Reproduction.

[61] Weir C.P., Robaire B. (2007). Spermatozoa Have Decreased Antioxidant Enzymatic Capacity and Increased Reactive Oxygen Species Production during Aging in the Brown Norway rat. J. Androl..

